# Novel Insights into Carbapenem Resistance: Mechanisms, Diagnostics, and Future Directions

**DOI:** 10.3390/antibiotics15030270

**Published:** 2026-03-05

**Authors:** Ionela-Larisa Miftode, Viorel Dragoș Radu, Raul-Alexandru Jigoranu, Daniela-Anicuța Leca, Cristian Sorin Prepeliuc, Maria Antoanela Pasare, Radu-Stefan Miftode, Maria Gabriela Grigoriu, Tudorița Gabriela Parângă, Egidia Gabriela Miftode

**Affiliations:** 1 Faculty of Medicine, “Grigore T. Popa” University of Medicine and Pharmacy, 700115 Iasi, Romania; ionela-larisa.miftode@umfiasi.ro (I.-L.M.); daniela.leca@umfiasi.ro (D.-A.L.); antoanela.pasare@gmail.com (M.A.P.); radu-stefan.miftode@umfiasi.ro (R.-S.M.); grigoriumaria.mg@gmail.com (M.G.G.); tudorita.paranga@umfiasi.ro (T.G.P.); egidia.miftode@umfiasi.ro (E.G.M.); 2 “St Parascheva” Clinical Hospital of Infectious Diseases, 700116 Iasi, Romania; 3 Department of Urology, “Dr. C.I. Parhon” University Hospital, 700503 Iasi, Romania

**Keywords:** carbapenem resistance, Enterobacterales, genome dynamics, heteroresistance, porin loss, antimicrobial stewardship

## Abstract

Carbapenems are essential for the treatment of severe infections caused by Gram-negative bacteria, particularly in critically ill and immunocompromised patients. However, the global rise of carbapenem-resistant Enterobacterales (CRE), *Pseudomonas aeruginosa*, and *Acinetobacter baumannii* has significantly eroded their effectiveness, and the phenomenon is now recognized as a major public health threat. Resistance is driven by the complex and evolving interplay of enzymatic and non-enzymatic mechanisms, occurring within highly successful clonal lineages and mobile genetic platforms. This review summarizes advances since 2020 in the molecular basis of carbapenem resistance, integrating enzymatic mechanisms across Ambler classes A, B, C, and D with emerging non-enzymatic contributors, including porin remodeling, efflux pump upregulation, target-site alterations, and outer-membrane adaptations. Particular attention is given to adaptive genome dynamics, such as IS26-mediated gene amplification, plasmid multimerization, and heteroresistance, that generate unstable resistance phenotypes and complicate routine susceptibility testing. Newly introduced β-lactam/β-lactamase inhibitor combinations exert distinct selective pressures: ceftazidime–avibactam favors KPC Ω-loop variants and permeability defects, often restoring carbapenem susceptibility, whereas meropenem–vaborbactam and imipenem–relebactam resistance is driven mainly by porin loss and β-lactamase gene amplification. Cefiderocol resistance is multifactorial, frequently involving impaired siderophore uptake and heteroresistance, while sulbactam–durlobactam remains active against OXA-producing *A. baumannii* but is compromised by metallo-β-lactamases and PBP3 alterations. Carbapenem resistance is increasingly characterized by convergent, multi-layered adaptations that undermine both established and novel therapies. While high-level randomized evidence remains limited for some resistance mechanisms, emerging mechanistic, microbiological, and clinical data support the need for mechanism-aware diagnostics, repeated susceptibility assessment during therapy, and stewardship strategies informed by resistance biology. Integrating molecular context into routine practice will be critical to preserving emerging treatment options and limiting the global impact of carbapenem resistance.

## 1. Introduction

Carbapenems are a critical line of defense against severe Gram-negative infections. Their robustness against extended-spectrum and AmpC β-lactamases, combined with optimal pharmacokinetic and pharmacodynamic properties, supports their use as last-resort agents in critically ill and immunocompromised patients [1,2,3].

However, surveillance data confirm that carbapenem resistance has reached alarming levels globally. According to the WHO Global Antimicrobial Resistance and Use Surveillance System (GLASS) 2025 report, the global median resistance across 93 pathogen–antibiotic combinations was 17.2%, with substantially higher regional medians in South-East Asia (31.1%) and the Eastern Mediterranean (30.0%). In bloodstream infections specifically, carbapenem resistance in *Acinetobacter* spp. reached 54.3% globally, while carbapenem-resistant *Klebsiella pneumoniae* reached 41.2% in the South-East Asia region [4]. Temporal analyses between 2018 and 2023 further demonstrated annual relative increases in carbapenem resistance of 12.5% in *Escherichia coli* and 15.3% in *K. pneumoniae* in certain regions. These data quantitatively frame the escalating burden of carbapenem-resistant Enterobacterales (CRE), *Pseudomonas aeruginosa*, and *Acinetobacter baumannii* [4]. In addition to resistance proportions, population-level incidence data further illustrate the clinical burden. According to ECDC surveillance, the incidence of carbapenem-resistant *K. pneumoniae* bloodstream infections in the EU/EEA reached approximately 3.97 cases per 100,000 population in 2023, representing a substantial increase compared with pre-pandemic levels [5]. Recent CDC analyses indicate that reported carbapenem-resistant infections increased from 1.98 to 3.16 cases per 100,000 persons in the U.S. from 2019 to 2023, driven in part by NDM-producing CRE, which rose ~460% in the same period [6]. ICU-based cohorts report incidence densities of carbapenem-resistant *A. baumannii* bacteraemia exceeding 5–10 cases per 1000 ICU admissions in high-burden settings [7].

In particular, carbapenem-resistant *A. baumannii* (CRAB) now accounts for a substantial share of antibiotic resistance-linked severity and mortality [8,9]. In CRAB, the international clone IC2 (corresponding to ST2) remains overwhelmingly dominant. One recent global analysis found that 43.7% of *A. baumannii* strains belonged to ST2 [10]. Moreover, in clinical isolates from a hospital in East Africa, ST2 CRAB was the most prevalent sequence type reported [9]. Other settings observed IC2 dominating intensive care unit (ICU) outbreaks, including clones co-producing OXA-23 and NDM-1 [3,11,12].

Within *K. pneumoniae*, clonal expansion has driven much of the problem. Surveillance from Southern Europe (2016–2018) identified the major high-risk clones, ST258/512, ST101, ST11, and ST307, as responsible for the majority of carbapenem-resistant *K. pneumoniae* (CRKP) infections, with region-specific patterns: KPC-associated ST258/512 in Greece, Italy, and Spain; OXA-48 in ST101 in Serbia/Romania; NDM in ST11 in Greece; and OXA-48–like ST14 in Turkey [1,13,14,15]. The ST307 clone, in particular, has garnered attention as a globally emerging high-risk lineage. It has been associated with outbreaks in Germany marked by extensive drug resistance and hypervirulence features, including siderophore expression and hypermucoviscosity [15]. Additionally, outbreaks of NDM-5–producing ST307 CRKP have been reported in Shanghai, China and Italy [16,17].

Among carbapenem-resistant *P. aeruginosa*, global high-risk clones such as ST235 are predominant. ST235 is particularly concerning as the most globally widespread clone, associated with over 60 acquired β-lactamase variants (including multiple carbapenemases) and increased virulence linked to higher mortality risk [18]. ST235’s success comes from a remarkable capacity for acquiring resistance genes and high adaptability in hospital settings [18]. These clones are presented as representative high-risk lineages that are increasingly prevalent and frequently associated with multidrug resistance and virulence traits, rather than as a comprehensive epidemiological overview.

The 2024 WHO Bacterial Priority Pathogens List ranks CRKP as the top global AMR priority, with other critical pathogens including *P. aeruginosa* and *Acinetobacter* spp. also featured prominently [19]. Likewise, the IDSA’s 2024 guidance on treating AMR Gram-negative infections specifically emphasizes CRE, CRAB, and difficult-to-treat resistant (DTR) *P. aeruginosa* as urgent threats. In addition, it highlights the need for the fact that management of these infections necessitates mechanism-informed therapeutic approaches [20].

**Figure 1:**
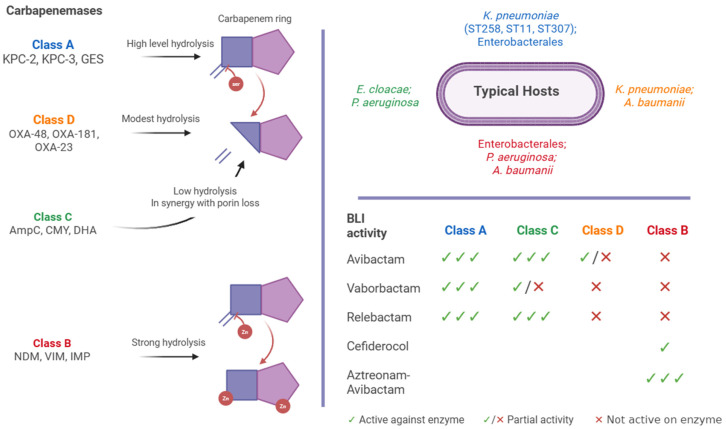
Major carbapenemases classified according to Ambler classes (A–D), showing representative enzymes, typical bacterial hosts, relative carbapenem hydrolytic activity, and BLI activity profiles. Arrows indicate the relative degree of carbapenem hydrolysis, and red “Zn” symbols denote zinc-dependent catalytic activity of class B MBLs. The BLI activity matrix summarizes representative inhibitory activity against each class based on published literature. Created in BioRender. Pasare, A. (2026); https://BioRender.com/wus6nas (accessed on 23 February 2026).

Beta-Lactamases are grouped into Ambler classes A, B, C, and D according to their amino acid sequences, and this classification is clinically relevant because it determines whether a β-lactamase inhibitor (BLI) can effectively restore antibiotic activity (Figure 1). Classes A, C, and D are serine β-lactamases that share a catalytic serine residue at the active site, whereas class B metallo-β-lactamases (MBLs) depend on zinc ions for their activity [21,22]. This distinction is particularly important when guiding empirical therapy: avibactam, vaborbactam, and relebactam are effective against many class A and C enzymes and exhibit partial activity against certain class D carbapenemases [23,24], but none of the licensed BLIs are active against class B MBLs [24]. As a result, infections caused by MBL-producing pathogens (e.g., NDM, VIM, IMP) must be approached with alternative agents such as cefiderocol, which bypasses hydrolysis via iron uptake pathways, or the investigational aztreonam–avibactam (ATM/AVI) combination, which couples a monobactam stable to MBL activity with potent inhibition of co-produced serine β-lactamases. Thus, the Ambler classification is not a theoretical framework but a practical tool that directly informs antimicrobial stewardship and guides bedside treatment selection [23,24].

These classes are defined as follows:

- Class A (KPC, some GES)—These enzymes are generally inhibited by BLIs such as avibactam (with ceftazidime), vaborbactam (with meropenem), and relebactam (with imipenem). These combinations remain powerful against *K. pneumoniae* carbapenemase producers, though emerging variants with Ω-loop mutations (e.g., KPC-41, KPC-50) can reduce efficacy [25,26,27].
- Class C (AmpC)—AmpC β-lactamases, whether chromosomal or plasmid-encoded, are efficiently inhibited by avibactam and relebactam, whereas vaborbactam’s activity is more variable [25,28].
- Class D (OXA-48-like)—Avibactam exhibits variable inhibitory activity across OXA-48-like enzymes, effective against some alleles (like OXA-48/181), with reduced or inconsistent inhibition observed in certain variants (e.g., OXA-244/232). Currently, neither vaborbactam nor relebactam is effective against these enzymes [26,29,30].
- Class B (MBLs: NDM, VIM, IMP)—Licensed BLIs do not inhibit MBLs. Therapeutic strategies instead rely on the following:
- Cefiderocol, a siderophore cephalosporin that bypasses enzymatic defenses via iron transport but is associated with emerging resistance and higher mortality in some contexts [31].
- ATM/AVI, which currently shows strong in vitro activity across MBL-producing CRE [31,32,33].

Therapeutic innovation continues. In 2024, the FDA approved cefepime–enmetazobactam (marketed as Exblifep) for the treatment of complicated urinary tract infections (cUTIs), including pyelonephritis, in adults, a significant advancement for targeting infections caused by extended-spectrum β-lactamase (ESBL), as well as AmpC-producing Gram-negative bacteria. Approval was based on robust clinical data from the global Phase III ALLIUM trial, which demonstrated both non-inferiority and superiority compared to piperacillin–tazobactam in terms of clinical cure and microbiological eradication [34]. Notably, the agent remains inactive against MBL-producing pathogens, highlighting the continued limitations of novel beta-lactam/beta-lactamase inhibitor (BL/BLI) combinations in covering class B carbapenemases [34,35,36,37,38].

Another recent advance is sulbactam–durlobactam (SUL-DUR–Xacduro), which received U.S. approval in 2023 for CRAB pneumonia. In the ATTACK Phase III trial, it was non-inferior to colistin, with 28-day mortality of 19% vs. 32.3%. Importantly, it also shows no activity against MBL-producing strains [39,40].

This narrative review synthesizes recent advances in carbapenem resistance among major Gram-negative pathogens. It integrates mechanistic, microbiological, and clinical evidence, linking enzymatic and non-enzymatic pathways with emerging genome-dynamic phenomena, diagnostic strategies, and therapeutic innovations. By situating molecular resistance mechanisms within evolving antimicrobial approaches, it aims to provide a coherent framework to inform clinical decision-making, stewardship, and surveillance.

## 2. Enzymatic Mechanisms—What’s New Since 2020

### 2.1. Class A Carbapenemases

The KPC family remains the most clinically consequential carbapenemase in *K. pneumoniae* and other Enterobacterales, but recent years have brought clear evidence that this enzyme continues to evolve under ceftazidime–avibactam (CZA) pressure. The most notable novelties are Ω-loop mutations at Ambler position 179 (especially D179Y and D179N), which have been associated with resistance to ceftazidime–avibactam. Structural studies demonstrate that these substitutions destabilize the Ω-loop and reduce avibactam binding affinity, thereby altering active-site dynamics [41]. In clinical isolates, variants at this position have been linked to ceftazidime–avibactam resistance and altered carbapenem susceptibility profiles [42].

Clinically, this leads to the “seesaw” resistance pattern: isolates harboring D179 variants exhibit high-level CZA resistance but restored susceptibility to carbapenems such as meropenem or to M/V. Retrospective clinical data demonstrate that KPC-2 D179Y mutants in ST258 strains often display a 2- to 9-fold reduction in meropenem MICs, enabling effective use of meropenem-containing regimens after CZA failure [43]. Moreover, global surveillance has catalogued over 190 distinct *bla_KPC_* subtypes, calling attention to the extensive adaptive landscape of these enzymes under therapeutic selection pressure [44].

Beyond KPC, under-detected carbapenemases, such as IMI, NmcA, FRI, SME, and certain GES variants, are quietly circulating across human, animal, and environmental reservoirs. Their detection is often missed by routine diagnostic workflows, posing both therapeutic and public health challenges [45]. Clinicians should be alert to unexplained carbapenem resistance, and laboratories may need to adopt expanded detection panels or sequencing-based platforms to identify these hidden threats.

### 2.2. Class B (MBLs)

Among MBLs, NDM has shown the fastest global spread, with at least 24 variants (NDM-1 to NDM-24), spreading across multiple Enterobacterales species worldwide [46,47]. Unlike other MBLs, NDM is a lipoprotein anchored to the outer membrane, a feature that enhances its stability under host-induced zinc starvation. Evolutionary analysis reveals that substitutions such as M154L improve Zn(II) binding affinity, enabling variants to maintain activity in low-zinc environments, thereby boosting bacterial fitness during infection [48]. Plasmid-mediated transfer is central to *bla*_NDM_ spread, with IncX3 plasmids driving *bla*_NDM-5_ in *E. coli* ST167 and novel IncHI5-like and IncI1 plasmids emerging as additional carriers; co-occurrence with *mcr* colistin resistance genes further complicates therapy [49].

VIM-type MBLs also continue to diversify, with novel alleles expanding both clinical and environmental reservoirs. VIM-92, identified in *P. aeruginosa*, was carried on a class 1 integron within the Tn1403 transposon on a megaplasmid, illustrating its high mobility in hospital settings [50]. Similarly, VIM-71, detected in *Vibrio alginolyticus* isolated from seafood, highlights the presence of MBL determinants in marine environments, which may serve as reservoirs for resistance genes capable of re-entering clinically relevant pathogens through horizontal gene transfer [51]. Together, these findings reinforce the interconnected clinical and environmental dimensions of MBL evolution.

IMP-type carbapenemases also show concerning trends. Certain variants display reduced susceptibility to xeruborbactam, a promising MBL inhibitor still in development. In addition, IMP-4 has recently been identified on conjugative plasmids in *Citrobacter youngae*, further extending the host range of this important resistance determinant [52].

Co-production of MBLs with ESBLs and AmpC β-lactamases is common, complicating detection and severely restricting treatment options. This is particularly concerning because MBL producers remain resistant to all currently approved β-lactamase inhibitors, rendering most BL/BLI regimens ineffective [53]. Consequently, cefiderocol has become critical. In the pivotal CREDIBLE-CR trial, cefiderocol achieved a 70.8% clinical cure rate against MBL-producing infections, outperforming standard comparators [54,55]. Still, emerging resistance threatens its efficacy, as treatment failures have been reported [56].

### 2.3. Class D Carbapenemases

Over the past few years, novel OXA-48-like variants have been unveiled, expanding our understanding of this enzyme family’s diversity [57]. For instance, two new alleles, *bla*_OXA-1038_ and *bla*_OXA-1039_ were discovered in *Shewanella xiamenensis*, both exhibiting significant carbapenem-hydrolyzing activity when expressed in *E. coli* and unaffected by classical BLI [58].

Clinically, OXA-48 producers pose unique challenges because they often confer only modest carbapenem MIC elevations, making them difficult to detect by routine phenotypic methods. They are typically resistant to ertapenem but may appear susceptible to imipenem or meropenem, leading to misclassification and therapeutic delay [59]

In parallel, surveillance data reveal a worrisome global rise in OXA-48-like prevalence among carbapenemase-producing Enterobacterales. In Taiwan, the incidence of OXA-48 rose six fold between 2012 and 2015, while US centers reported an increase from 0.3% in 2019 to 8.2% in 2021, signaling sustained challenges in detection and infection control [60].

To provide a quantitative framework for the enzymatic mechanisms discussed above, Table 1 summarizes the distribution of major carbapenemase classes across key Gram-negative pathogens, integrating recent evolutionary variants with reported prevalence in patient populations and associated clinical outcomes.

## 3. Permeability, Efflux, and Target Alterations

### 3.1. Porins in Enterobacterales

**Figure 2:**
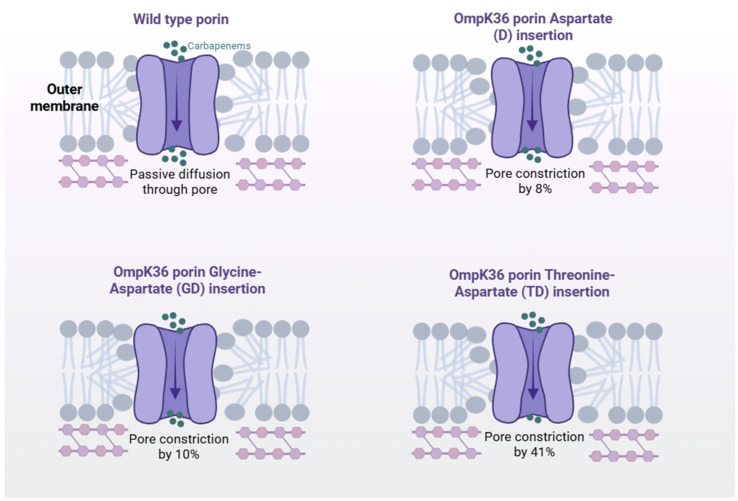
Conceptual schematic of OmpK36 loop 3 (L3) insertions and their predicted effect on porin channel diameter in *Klebsiella pneumoniae*. The wild-type porin permits passive diffusion of carbapenems. Single–amino acid insertions within loop 3—Aspartate (D), Glycine-Aspartate (GD), and Threonine-Aspartate (TD)—result in progressive pore constriction (approximately 8%, 10%, and 41%, respectively), as reported in structural-genomic analyses. Arrows indicate reduced antibiotic influx through the narrowed channel. Created in BioRender. Pasare, A. (2026) https://BioRender.com/f7yst2x (accessed on 23 February 2026).

Recent global structural-genomic analyses have highlighted that OmpK36 loop 3 (L3) insertions, including D, GD, and TD variants, are prevalent across *K. pneumoniae* high-risk clones, causing substantial porin constriction and elevated carbapenem MICs. Notably, the TD insertion reduces porin’s size by approximately 41%, far more than GD (10%) or D (8%) variants, and all three insertions significantly increase meropenem resistance, especially when paired with KPC or chromosomal AmpC β-lactamases (Figure 2) [63].

These OmpK36 L3 variants also influence the performance of novel BL/BLI combinations. An experimental study revealed that M/V and CZA combinations exhibit differential in vitro synergy depending on the specific porin mutation background, reinforcing the therapeutic significance of porin genotype [64,65].

Further confirming clinical relevance, case reports describe the in vivo selection of OmpK36-deficient subpopulations during antibiotic therapy, illustrating that porin-mediated resistance evolves under treatment pressure [66]. Moreover, OmpK35 inactivation (often through truncation) combined with OmpK36 L3 insertions is increasingly linked to reduced susceptibility to cefiderocol in certain genetic contexts. Although this mechanism is recognized, specific mechanistic data are still emerging, pointing to areas ripe for further investigation.

### 3.2. Pseudomonas aeruginosa

Nationwide genomic surveillance of carbapenem-resistant *P. aeruginosa* (CRPA) has reinforced that OprD loss or downregulation remains the principal driver of imipenem resistance. However, these datasets also uncovered that 4.7% of CRPA isolates carry mutations in *ftsI* (encoding penicillin-binding protein (PBP)-3), marking a notable addition to the resistance repertoire https://journals.asm.org/doi/10.1128/mbio.01343-25?utm_source=chatgpt.com (accessed on 6 September 2025) [67].

In *P. aeruginosa*, OprD loss remains the dominant, imipenem-specific permeability lesion, so when genomics/phenotype point to OprD inactivation, imipenem (±relebactam) is intrinsically disadvantaged and alternatives should be prioritized [68]. Emerging PBP3 (*ftsI*) substitutions measurably erode carbapenem activity and can shift relative performance within the β-lactam class (e.g., some variants depress carbapenems while leaving cefiderocol or piperacillin less affected), arguing for mechanism-aware drug selection rather than a generic “escalate the β-lactam” approach [69,70]. Upregulation of resistance–nodulation–cell division (RND) efflux systems (MexAB-OprM and MexXY), often accompanied by mutations in regulatory genes, represents a common pathway to non-carbapenemase resistance in carbapenem-resistant *P. aeruginosa*. These mechanisms can reduce susceptibility to newer BL/BLI regimens. Therefore, when selecting among agents such as ceftolozane–tazobactam, ceftazidime–avibactam, imipenem–relebactam, or cefiderocol, phenotypic susceptibility testing should be interpreted alongside available genotypic resistance data [71,72].

The new untargeted-metabolomics map of Mex natural substrates (QS signals, phenazine by-products, oxidized fatty acids) provides concrete chemotypes to develop competitive efflux-pump inhibitors (EPIs) that could both potentiate antibiotics and attenuate virulence, an approach long proposed but now more rationally tractable (even if no EPI is yet in human trials) [73,74]. Finally, high-frequency within-host sequencing shows these permeability/efflux/target lesions can variate within days under therapy, so repeat AST/genomics during prolonged treatment is justified to catch reversions or new resistance paths and to adapt therapy dynamically [75].

### 3.3. Acinetobacter baumannii

Beyond the well-established role of CarO porin loss, emerging mechanistic studies suggest that outer membrane remodeling, including alterations in OmpA-like proteins such as YiaD, may affect envelope integrity and potentially influence β-lactam entry [76]. These findings broaden the current understanding of permeability-associated resistance beyond simple porin disruption. Recent functional studies of OmpA-like outer membrane proteins demonstrate that envelope remodeling can influence antibiotic penetration, bacterial physiology, and resistance phenotypes [77]. However, the clinical relevance and quantitative contribution of these membrane changes to carbapenem resistance remain incompletely defined. While CarO disruption continues to represent a primary mechanism, additional envelope alterations may modulate antibiotic penetration and susceptibility in specific contexts.

In *A. baumannii*, the dominant resistance axis is still OXA-type carbapenemases driven by ISAba1 promoters, which means that mechanism-aware therapy matters: when genomic testing shows *bla*_OXA-23/-51/-58_ with upstream ISAba1, SUL-DUR becomes the preferred β-lactam option because durlobactam potently inhibits class D enzymes while sulbactam restores PBP targeting; this was the first regimen to show efficacy in a CRAB-focused phase 3 trial and is now FDA-approved for HAP/VAP due to susceptible ABC organisms [78,79].

By contrast, co-production of OXA with NDM-1 is increasingly reported and further collapses β-lactam choices; in these settings, clinicians often pivot to cefiderocol, for which observational syntheses suggest outcome advantages over colistin-based therapy, even though heteroresistance and on-therapy resistance via siderophore-uptake lesions remain real risks that argue for close MIC/genomic follow-up [80,81]. Envelope factors are not just academic: porin/envelope remodeling (e.g., CarO variants and OmpA-like proteins such as YiaD) can blunt β-lactam entry and modulate meropenem response, while also shaping virulence, supporting concurrent susceptibility testing (including cefiderocol where appropriate) and opening the door to emerging anti-OmpA/OMV-based adjuncts in difficult CRAB [77].

Importantly, a 2024 clinical study demonstrated that *bla*_OXA-23_ copy-number amplification does not necessarily correlate with increased carbapenem MICs, challenging the assumption that gene dosage alone equates to a stronger resistant phenotype [78]. Moreover, the active mobilization of the chromosomal *bla*_OXA-23_ gene by mobile genetic elements, such as transposons (Tn2006–Tn2009) and conjugative plasmids, continues to be detected in recent *A. baumannii* isolates. This highlights the organism’s remarkable genomic flexibility, which drives resistance evolution and facilitates the horizontal spread of key resistance genes [82].

## 4. Gene Dosage and Genome Dynamics

### 4.1. IS26-Driven Amplification and Heteroresistance

Across Enterobacterales, the insertion sequence IS26 frequently builds translocatable units (TUs), which are mobile genetic segments that capture β-lactamase genes and can multiply in tandem on plasmids or chromosomes. Long-read sequencing shows that these TUs often form long head-to-tail chains flanked by IS26, sometimes reaching dozens of copies. As copy number increases, sequencing read depth, gene expression, and carbapenem MICs rise proportionally.

These arrays are highly dynamic. Under antibiotic pressure, tandem copies can expand rapidly through conservative transposition and replicative stacking; when selective pressure is withdrawn, copy numbers may contract. This reversible amplification produces fluctuating resistance levels without requiring stable point mutations.

Such IS26-mediated copy-number plasticity represents a major molecular driver of the heteroresistance phenomenon discussed in Section 5.1. Only a minor subpopulation carrying high TU copy numbers may survive initial antimicrobial exposure, yet this fraction can rapidly dominate under continued carbapenem pressure.

Other duplication routes, such as those involving ISEcp1, have also been described, although IS26-mediated TUs appear to predominate in clinical CRE. Clinically, such copy-number variability may help explain discordant susceptibility results, for example, when PCR detects a resistance gene but phenotypic testing underestimates its expression level, or when isolates that appear susceptible after subculture regain higher MICs during therapy. However, while amplification-driven heteroresistance has been associated with adverse therapeutic outcomes, direct prospective evidence linking IS26-mediated amplification alone to treatment failure remains limited [83].

Recent population-level and single-isolate studies extend this principle: copy-number flexibility is now recognized as a general route to β-lactam heteroresistance continua, with dynamic amplification of pre-existing β-lactamases shifting MIC distributions during treatment. This favors quantitative diagnostics (read-depth whole-genome sequencing (WGS), ddPCR/qPCR) and population-based AST when treatment decisions hinge on narrow MIC margins [84].

### 4.2. Plasmid Multimerization as a Dosage Rheostat

Carbapenemase levels can also rise through plasmid multimerization, a mechanism whereby tandem copies of resistance plasmids increase gene dosage under antibiotic pressure. In a model involving the *bla*_IMP-6_ gene, exposure to meropenem promoted the formation of multimeric versions of the IncN plasmid carrying *bla*_IMP-6_. This increased plasmid copy number, mRNA expression, and meropenem MICs. When antibiotic pressure was removed, the plasmids reverted toward their normal monomeric state. At the molecular level, recombination and rolling-circle replication generate concatemers (plasmid copies linked end-to-end) that segregate inefficiently during cell division. This temporarily raises the number of *bla*_IMP-6_ copies per cell, producing reversible spikes in resistance that standard presence/absence assays fail to detect [85]. Although plasmid multimerization was demonstrated in clinical CRE isolates [85], available data derive primarily from single-center surveillance and mechanistic analyses.

More broadly, recent work demonstrates that copy-number flexibility itself constitutes a generalizable adaptive strategy, enabling bacteria to dynamically amplify resistance determinants in response to escalating antibiotic pressure while mitigating long-term fitness costs through compensatory mechanisms [84].

Robust epidemiologic data quantifying the frequency of plasmid multimerization in clinical carbapenem-resistant Enterobacterales are currently lacking, as most surveillance frameworks prioritize plasmid incompatibility groups and transmission dynamics rather than structural plasmid rearrangements. Nonetheless, plasmids remain the principal vehicles of carbapenemase dissemination, with broad-host-range replicons (e.g., IncN, IncL/M, IncF, IncX3) driving interspecies spread and shaping resistance phenotypes during healthcare-associated outbreaks [86,87].

### 4.3. Transposons & Plasmid Backbones

Tn4401, a member of the Tn3 transposon family, remains the archetypal carrier of *bla*_KPC_. Its various isoforms, defined by promoter differences and IS-mediated truncations, modulate both *bla*_KPC_ expression and mobility. KPC genes occur across several plasmid backbones (e.g., IncFII_K_, IncN, IncI2, IncX, IncA/C, IncR), and these replicon contexts influence plasmid stability, copy number regulation, and co-selection dynamics [88]. For OXA-48-like enzymes, the IncL plasmid remains the globally dominant and remarkably stable vector, accounting for the efficient endemic spread and persistence of OXA-48-like producers in colonization reservoirs [89].

In contrast, the dissemination of NDM carbapenemases has diversified. While early transfers were mediated by Tn125, more recent events involve IS26-flanked pseudo-composite transposons and Tn3000, which are often embedded in nearly identical IncX3 plasmids. These compact backbones readily move *bla*_NDM-1_ and *bla*_NDM-5_ across species with limited fitness cost. Moreover, they frequently carry additional AMR determinants, forming co-resistance platforms that enhance persistence and effectively increase the “dosage” of carbapenem resistance [90,91].

### 4.4. Integrative Elements Carrying IMI/NmcA in Enterobacter

In *Enterobacter* spp., IMI/NmcA carbapenemases are typically chromosomally encoded within XerC/XerD-dependent integrative mobile elements (IMEX), often referred to as EcloIMEX. These elements insert at a conserved setB–yeiP locus and can form circular intermediates that, in principle, enable mobilization. However, they primarily confer vertical stability and consistent gene expression, contrasting with the fluctuating “copy-number bounces” seen in plasmid-mediated systems. Recent clinical and environmental surveys continue to detect IMI/NmcA and related FRI enzymes across hospital, wastewater, and animal settings, emphasizing their One Health significance. These findings highlight the need for sequence-resolved diagnostics capable of identifying not just the presence of resistance genes but also their genetic context and element architecture [92].

### 4.5. Practical Diagnostics & Therapy Implications

It is not sufficient to rely solely on gene detection when assessing carbapenem resistance. Both the copy-number state (amplified versus baseline) and the genetic vehicle (e.g., IS26-mediated translocatable unit versus single chromosomal copy, multimerized plasmid versus monomer) directly influence MICs and the likelihood of heteroresistance. Routine PCR should therefore be complemented by read-depth-based WGS or quantitative (dd)PCR in complex or borderline cases, and population-level analyses considered when MICs hover near clinical breakpoints.

Resistance phenotypes should not be assumed stable over time. Amplification-driven resistance can collapse after subculture but rapidly re-expand during antibiotic exposure. Repeat AST and genomic testing during prolonged therapy can help detect these dynamic shifts and prevent treatment failure [83].

Finally, the backbone context remains critical. The intrinsic stability of IncL plasmids (carrying OXA-48-like enzymes) supports long-term persistence and colonization, whereas IncX3 (NDM) and multi-resistance IncF/A/C plasmids promote co-selection and dissemination. These backbone-specific traits should inform infection control and antimicrobial stewardship strategies, including approaches to limit collateral selection pressure [89].

## 5. Mechanistic Insights from Phenotypes

### 5.1. Heteroresistance and the Inoculum Effect as Genome-Dynamic Phenotypes

Heteroresistance refers to the coexistence, within a genetically related bacterial population, of subpopulations displaying substantially higher MICs than the dominant phenotype, despite routine susceptibility testing categorizing the isolate as susceptible. Unlike stable resistance caused by fixed mutations, heteroresistance is often reversible and reflects dynamic genomic processes that alter gene dosage, expression, or permeability under antibiotic pressure [93,94].

In carbapenem-resistant Gram-negative pathogens, heteroresistance most commonly arises from gene amplification events, particularly those involving *bla*_KPC_ and related carbapenemase genes, often mediated by mobile elements such as IS26. Expansion of tandem transposition units increases enzyme copy number under carbapenem exposure and contracts when selective pressure is removed, producing fluctuating MIC profiles [94,95]. Additional contributors include plasmid copy number variation and permeability modulation, each capable of generating resistant subpopulations without permanent genetic fixation [93].

The carbapenem inoculum effect (IE), defined as a significant MIC increase at higher bacterial inocula, represents a related phenotypic manifestation of these mechanisms. Amplified carbapenemase genes, permeability bottlenecks, or efflux overexpression can generate enzyme saturation dynamics that become apparent only at higher bacterial densities. Mechanistic studies demonstrate that carbapenemase producers typically exhibit a pronounced IE compared with isolates whose resistance is primarily driven by porin loss alone [96,97].

Clinically, heteroresistance and the carbapenem inoculum effect explain discordant AST results and treatment failures in isolates categorized as “susceptible by standard AST.” Amplification-driven carbapenemase expression, plasmid copy-number shifts, and permeability modulation generate dynamic MIC fluctuations, particularly near clinical breakpoints [93,94,95,96,97]. Rather than laboratory artifacts, these phenotypes represent readouts of genome plasticity under antibiotic pressure. When MICs fluctuate or approach decision thresholds, repeat AST, higher inoculum testing, or population analysis may be warranted.

Conceptually, heteroresistance and the IE should be understood not as isolated laboratory artifacts but as phenotypic readouts of genome plasticity under antibiotic selection—a unifying principle that links gene amplification, plasmid dynamics, permeability remodeling, and treatment-emergent resistance, as discussed throughout this review.

### 5.2. Practical Lab Algorithms When WGS Is Not Available

The interpretive patterns described below are based on a synthesis of published microbiological and clinical data and are intended as a practical framework rather than formal consensus guidelines.

Because ertapenem is more susceptible to the effects of porin loss and ESBL/AmpC backgrounds than imipenem or meropenem, a pattern of disproportionately elevated ertapenem MICs with lower meropenem or imipenem values typically indicates permeability defects, with or without ESBL/AmpC activity. In contrast, uniform elevation of meropenem and imipenem MICs, sometimes accompanied by an inoculum effect, is more suggestive of a carbapenemase mechanism. Classic genetic complementation restoring OmpK36 function in *Klebsiella* markedly reduces ertapenem MICs and restores susceptibility to imipenem and meropenem, and outbreaks of ertapenem-resistant, ESBL-positive *K. pneumoniae* have been consistently linked to OmpK36 variants [98,99].

When WGS is not available, contemporary phenotype-first diagnostic algorithms remain effective for resistance mechanism inference. Initial screening should employ ertapenem, the most sensitive carbapenem for early detection, followed by confirmation with a second carbapenem such as meropenem or imipenem. Subsequent carbapenemase activity assays, including the modified carbapenem inactivation method (mCIM), Carba NP, and CIM variants, or inhibitor-based disc tests are then used to distinguish among class A, B, and D enzymes [100,101]. Where accessible, targeted PCR panels can provide additional resolution [102].

In cases of discordant results, for example, when an inoculum effect is observed but carbapenemase activity tests are negative, resistance should be triaged toward porin loss or AmpC-mediated mechanisms, with possible contributions from copy-number variation and heteroresistance also considered [83]. Low-cost diagnostic workflows that combine stepwise phenotypic testing with selective confirmatory assays have shown reliable performance for both common carbapenemases and weakly hydrolyzing enzymes [101].

At the bench, several interpretive cues can strengthen this approach. Heteroresistance or an IE should prompt suspicion of gene amplification or high-level enzyme activity; repeating AST with population analysis or higher inocula may reveal hidden resistance [96]. A disproportionately elevated ertapenem MIC relative to meropenem typically signals a permeability bottleneck, often from OmpK36 loss combined with ESBL/AmpC activity, which can be further confirmed by inhibitor discs or porin immunoblots [103]. Conversely, uniform elevation of all carbapenem MICs with a positive carbapenemase test strongly supports enzyme production and should lead directly to class assignment—KPC (class A), MBLs (class B), or OXA-48-like enzymes (class D)—to guide rational use of BL/BLI combinations or chelator-based therapies (Table 2) [101].

## 6. Mechanisms Under Selection by New Therapies

The past decade has seen the rollout of several novel BL/BLI combinations and carbapenem-sparing agents that have reshaped the management of carbapenem-resistant Gram-negative infections. While these therapies, such as CZA, M/V, IMI/REL, ATM/AVI, cefiderocol, cefepime–taniborbactam, and SUL-DUR, initially restored activity against major resistance determinants, clinical use has been rapidly followed by reports of on-therapy resistance emergence.

Selection pressures under these new regimens commonly exploit a limited but convergent set of mechanisms: target-site remodeling of β-lactamases, amplification or overexpression of resistance genes, porin loss and permeability bottlenecks, efflux pump upregulation, and modifications of PBPs. Importantly, many of these adaptive pathways not only compromise the index drug but also generate cross-resistance across the BL/BLI class or to alternative carbapenem-sparing agents.

### 6.1. Ceftazidime–Avibactam (CZA)

Resistance to CZA in KPC-producing *K. pneumoniae* most commonly arises through Ω-loop mutations. Recent reports describe variants such as KPC-190 and KPC-228 emerging during therapy, often in high-risk ST11 backgrounds, and frequently associated with porin loss or IS26-flanked mobilization [104,105]. These variants confer high-level CZA resistance while sometimes restoring carbapenem susceptibility (“seesaw” phenotype), with variable mortality signals reported in clinical series [104]. Additional studies have identified convergent mechanisms including NDM production, efflux activation, and co-produced β-lactamases (e.g., AFM-2, PER-1) contributing to CZA failure in both CRKP and CRPA [106].

The clinical implications are significant: resistance can evolve during ongoing therapy, driven by a combination of enzyme remodeling and permeability defects. This highlights the need for repeat susceptibility testing during treatment and the importance of mechanism-guided therapeutic adjustments whenever CZA failure is detected.

### 6.2. Meropenem–Vaborbactam (M/V) and Imipenem–Relebactam (IMI/REL)

Resistance to M/V and IMI/REL in KPC-producing *K. pneumoniae* is driven far more by permeability changes than by target (enzyme) mutations, with truncation or remodeling of OmpK36, and frequently concurrent amplification of *bla*_KPC_, emerging as the dominant pattern. A 2024 molecular study of non-MBL KPC-*K. pneumoniae* with elevated M/V MICs confirmed both porin disruption (truncated/altered OmpK36) and increased *bla*_KPC_ copy number as key determinants of M/V non-susceptibility [107].

Consistent with this, experimental selection work showed that resistance arising under M/V pressure commonly entails OmpK36 defects plus stepwise increases in *bla*_KPC_ copy number, reinforcing the central role of permeability and gene dosage rather than inhibitor escape [108]. Parallel observations for IMI/REL indicate that selected resistant mutants typically retain OmpK36 alterations while KPC active-site changes are comparatively infrequent, again pointing to porin loss and copy-number effects as the primary route to failure [108,109].

Additional clinical and genomic series have documented the same convergence, resulting in OmpK35/36 disruption with increased *bla*_KPC_ copy number, which together produce cross-resistance patterns to both M/V and IMI/REL [110].

Clinical implications: when M/V or IMI/REL activity wanes during therapy, reassessment should include tests that capture functional porin expression and *bla*_KPC_ copy-number changes (or proxies), because cross-resistance can emerge even in the absence of new KPC mutations, and timely mechanism-informed regimen changes are warranted [111].

### 6.3. Aztreonam–Avibactam (ATM/AVI)

ATM/AVI pairs an MBL-stable monobactam with a non-BLI of ESBLs, AmpC and KPC, thereby restoring β-lactam activity against MDR Gram-negative pathogens, including MBL-producing Enterobacterales. Large surveillance cohorts demonstrate near-universal in vitro activity against Enterobacterales, NDM/VIM/IMP producers included, while activity against MBL-positive Pseudomonas is limited. Clinically, phase 3 REVISIT and ASSEMBLE trials report efficacy and acceptable safety in cIAI, cUTI and HAP/VAP. Regulatory status differs by region: ATM/AVI received EU authorization following an EMA positive opinion, whereas U.S. FDA approval was granted subsequently, with recommended dosing built around extended infusions. Collectively, these data position ATM/AVI as a key MBL-active option and a more streamlined alternative to CZA plus aztreonam co-administration [112].

Emerging resistance to ATM–AVI among Enterobacterales has been linked to multiple mechanisms. Structural alterations in PBP3, particularly four-amino-acid insertions near the β-lactam binding pocket, can reduce drug binding and contribute to elevated MICs. More importantly, plasmid-mediated AmpC β-lactamases, especially CMY variants such as CMY-42 and its close derivatives (CMY-141, CMY-145, CMY-146), have been strongly associated with decreased susceptibility; in recent surveillance, over 70% of ATM/AVI–nonsusceptible *E. coli* carried CMY enzymes. Increased expression or amplification of CMY genes, often due to higher plasmid copy numbers, has also been shown to exacerbate resistance [113].

ATM/AVI combines aztreonam stability to MBL hydrolysis with avibactam inhibition of co-produced serine β-lactamases. Reduced susceptibility has been associated with PBP3 insertions (e.g., YRIK/YRIN motifs), plasmid-mediated CMY variants with increased expression, and combined ESBL or AmpC overproduction with porin alterations, frequently acting synergistically in *E. coli* and *Enterobacter* spp. [29,113,114,115,116].

ATM/AVI (Emblaveo) received FDA approval in February 2025 for treating complicated intra-abdominal infections (cIAIs) in adults with limited or no alternative options, particularly targeting infections caused by Gram-negative pathogens including those producing MBLs, making it a key therapeutic advance in the fight against MBL-mediated resistance (Table 3) [117].

### 6.4. Cefiderocol

While not a carbapenem, cefiderocol serves as a critical carbapenem-sparing option, frequently deployed in similar clinical contexts. Emerging resistance to cefiderocol is increasingly linked to complex, multifactorial pathways. In a landmark in vitro resistance development study, Kriz et al. exposed *E. coli*, *K. pneumoniae*, *P. aeruginosa*, and *A. baumannii* strains to escalating cefiderocol pressure and identified 42 distinct mutations across 26 genes, including loss-of-function changes in tonB, cirA, and envZ, which collectively reduce siderophore-mediated drug influx [118]. Complementary resistance mechanisms emerged in the form of efflux-associated gene alterations (*baeS, czcS, nalC*), β-lactamase regulatory or inactivating mutations (*ampR, dacB*), and even PBP target site mutations (*mrcB*) [118].

In vivo evidence has surfaced as well: Morosi et al. documented a clinical *A. baumannii* isolate pair differing only by a novel chromosomal premature stop codon in a TonB-dependent receptor homolog, which markedly diminished cefiderocol uptake, demonstrated via mass spectrometry, and led to treatment-emergent resistance [119].

Moreover, Shields et al. reported heteroresistant *A. baumannii* isolates harboring tonB-dependent receptor gene lesions, which correlated with cefiderocol treatment failures (81% vs. 55% in non-heteroresistant cases) [120]. Additional surveillance in *P. aeruginosa* identified tonB-dependent receptor mutations in up to 25% of isolates, many predating cefiderocol exposure, and these were enriched in heteroresistant subpopulations on population analysis profiles [121]. Experimental mutagenesis further suggests that resistance often requires coordinated chromosomal and enzymatic changes rather than single-step mutations [44].

Clinical implications: These findings indicate that cefiderocol resistance often results from synergistic mechanisms, including impaired siderophore-mediated uptake, efflux changes, β-lactamase modifications, and PBP alterations. The risk of in vivo heteroresistance emergence during therapy necessitates vigilant susceptibility testing, cautious monotherapy use, and consideration of combination regimens or alternative agents when resistance is suspected.

### 6.5. Cefepime–Taniborbactam (FEP/TAN)

FEP/TAN is among the most promising broad-spectrum BL/BLI combinations in late-stage development. Taniborbactam is a bicyclic boronate inhibitor with activity against Ambler class A (including KPC), class C (AmpC), class D (including OXA-48-like), and several class B MBLs, notably NDM and VIM enzymes [122,123]. Unlike avibactam, which does not inhibit MBLs, taniborbactam directly engages the MBL active site via boronate–zinc coordination, thereby extending inhibitory coverage beyond serine carbapenemases. In vitro surveillance demonstrates potent activity against KPC-, OXA-48-like-, and many NDM-producing Enterobacterales. However, MBL inhibition is variant-dependent. Reduced susceptibility has been reported in isolates carrying NDM-9, NDM-30, and VIM-83, likely reflecting structural alterations within the enzyme active site that diminish inhibitor binding efficiency [122,124,125,126]. These findings highlight that FEP/TAN’s MBL coverage is not uniform across all variants. Additional resistance contributors include porin loss, efflux upregulation, and modified PBPs, although these factors often act in synergy with β-lactamase-mediated resistance rather than independently [126,127,128].

Mechanistically, FEP/TAN differs from ATM/AVI. Whereas ATM/AVI bypasses MBL activity by using an intrinsically MBL-stable monobactam scaffold (aztreonam) combined with serine β-lactamase inhibition, FEP/TAN relies on direct MBL inhibition. This distinction has clinical implications: ATM/AVI activity is less sensitive to structural variation within the MBL active site, while FEP/TAN efficacy may be more vulnerable to mutation-driven changes in MBL catalytic efficiency or inhibitor affinity. Consequently, in regions with high prevalence of diverse or emerging NDM variants, mechanism-aware diagnostics will be essential to guide optimal agent selection [127].

Clinical implication: Despite its impressive in vitro potency, the emergence of NDM variants with enhanced activity raises concerns. These observations bring out the necessity for proactive resistance surveillance, even before FEP/TAN becomes clinically available, to inform therapeutic use and mitigate the risk of resistance-driven treatment failures.

### 6.6. Sulbactam–Durlobactam (SUL-DUR)

SUL-DUR became the first pathogen-targeted therapy for CRAB with US FDA approval in May 2023, pairing sulbactam’s direct anti-*Acinetobacter* PBP activity (notably PBP3) with durlobactam, a diazabicyclooctane inhibitor of class D (e.g., OXA-23, OXA-24/40, OXA-58) and selected class A β-lactamases. Its registrational, pathogen-specific phase-3 ATTACK trial showed that SUL-DUR (with background imipenem–cilastatin) achieved higher clinical cure and substantially lower nephrotoxicity than colistin (also with background imipenem–cilastatin) in serious CRAB infections, establishing clinical effectiveness and safety in a randomized setting [39,129].

Mechanistically, resistance may be driven by MBL production or PBP3 substitutions [130,131]. First, MBLs and PBP3 alterations are the best-supported causes of decreased SUL-DUR susceptibility. Second, high-level OXA expression, often ISAba1-driven, may shape resistance burden. Moreover, while promoter hijacking by ISAba1 upstream of *bla*_OXA-23_ is a long-recognized engine of carbapenem resistance and high carbapenem MICs in *A. baumannii*, its epidemiologic ubiquity remains relevant context when interpreting SUL-DUR performance in OXA-rich lineages (i.e., strains with very high OXA expression burden), even though PBP3 changes and MBLs appear more determinative for SUL-DUR specifically [132]. Third, broader *Acinetobacter* resistance biology (other resistance pathways, including efflux pump upregulation) may modulate β-lactam exposure; these pathways are well documented in CRAB and are discussed in recent clinical and mechanistic reviews, though direct, isolate-level links to SUL-DUR failure are less consistent than for PBP3 and MBLs [133,134].

Clinically, post-approval evidence and case-based reports reinforce SUL-DUR’s utility against difficult CRAB phenotypes, including strains non-susceptible to colistin or cefiderocol, while reminding that on-therapy selection is possible in high-burden settings, hence the interest in judicious combination strategies and local stewardship. Reviews synthesizing ATTACK [35] plus real-world experience also outline where combination therapy (e.g., with cefiderocol) is being evaluated to suppress on-therapy resistance and cover polymicrobial backgrounds (Table 4) [133,135,136].

#### Carbapenem-Sparing Tetracyclines

Eravacycline is a validated carbapenem-sparing tetracycline derivative with clinical evidence for complicated intra-abdominal infections, and it retains activity against many MDR Enterobacterales, including subsets of CRE [136]. In contrast, doxycycline has unpredictable activity against CRE and should be considered only as a niche oral carbapenem-sparing option for uncomplicated CRE cystitis when in vitro susceptibility is documented [137]. Major guidance documents primarily frame doxycycline as low-evidence/limited-indication therapy (rather than a standard CRE agent), reinforcing restriction to carefully selected low-risk scenarios [20]. Recent multicenter retrospective data suggest doxycycline may achieve acceptable short-term outcomes in selected UTI patients lacking oral options, but the evidence remains small and non-comparative [138].

## 7. Diagnostic Implications (Mechanism-Aware)

Carbapenem resistance management increasingly depends on mechanism-resolved diagnostics rather than carbapenem MICs alone [20]. Because therapeutic efficacy of modern BL/BLI combinations, siderophore cephalosporins, and emerging inhibitors is highly mechanism-dependent, rapid differentiation of serine carbapenemases, MBLs, porin-driven resistance, and gene amplification has become clinically decisive [20]. Consequently, diagnostic workflows must evolve from binary carbapenemase detection toward quantitative, mechanism-aware profiling [21,100].

Phenotype-first carbapenemase screens remain foundational, but assay performance varies by enzyme class, expression level, and organism background. Carba NP provides results in approximately 2 h, with reported sensitivity of 90–100% for KPC and NDM producers but reduced sensitivity for OXA-48-like enzymes (as low as 60–80% in some evaluations), particularly when hydrolytic activity is weak or enzyme expression is low [139,140,141,142]. False negatives are more common in isolates with borderline carbapenem MICs or low carbapenemase production, and inter-laboratory variability reflects differences in inoculum density, substrate preparation, and interpretation thresholds.

The modified carbapenem inactivation method (mCIM), paired with EDTA-modified CIM (eCIM), offers high sensitivity (>95%) for Enterobacterales and enables phenotypic differentiation between serine carbapenemases and MBLs. However, sensitivity in *P. aeruginosa* is more variable (reported 80–95%), partly due to permeability barriers and efflux influencing carbapenem diffusion during testing [100,143,144]. Extended incubation improves detection of low-level producers but delays reporting.

Inhibitor-based disc tests (boronic acid for KPC/AmpC; EDTA or dipicolinic acid for MBLs) rely on zone-diameter shifts ≥ 5 mm, yet interpretation depends on baseline MIC, co-produced enzymes, and expression levels. These assays may misclassify isolates co-producing KPC and MBL or those with porin loss masking inhibitor synergy [21,145].

For rapid antigen detection, lateral-flow immunoassays (LFIAs) such as NG-Test CARBA 5 achieve reported sensitivity and specificity exceeding 95% for major carbapenemases and provide results within 15 min from colonies. However, rare false negatives occur with uncommon variants or low-expression OXA-48-like enzymes, and off-target faint bands have been described, requiring confirmatory testing in ambiguous cases [146,147,148]. In *A. baumannii*, species-specific assays (e.g., RESIST ACINETO) improve detection of OXA-23/24/58 and NDM directly from positive blood cultures, reducing turnaround time by 24–48 h compared with conventional workflows [149].

Low-level OXA-48-like producers frequently yield weak or negative Carba NP results due to limited hydrolytic activity and, in some strains, meropenem β-lactone formation that alters substrate availability. Combining an antigen-based assay or targeted PCR with mCIM increases overall diagnostic sensitivity and reduces false-negative risk [150,151,152].

Beyond qualitative detection, genomic tools introduce quantitative resolution. WGS read-depth normalization and digital droplet PCR (ddPCR) allow estimation of *bla* gene copy number. Copy-number increases have been associated with stepwise elevation of M/V MICs and with heteroresistant subpopulations detectable only under high inoculum conditions [153]. Similarly, increased *bla*_PER-1_ copy number correlates with higher cefiderocol MICs in *P. aeruginosa*, illustrating gene dosage effects on siderophore cephalosporin susceptibility [154].

Despite these advances, quantitative thresholds for clinically significant amplification are not standardized. Variability in sequencing depth, normalization algorithms, and reference genome selection limits cross-laboratory reproducibility. Moreover, susceptibility breakpoints remain phenotype-based and do not account for gene dosage, promoter variants, or porin disruption, creating discordance between genotype and observed MIC.

WGS additionally supports high-resolution outbreak mapping and detection of cryptic transmission clusters. In tertiary-care settings, real-time genomic surveillance has reduced time to cluster identification by several days compared with conventional epidemiology, but cost, turnaround time, and bioinformatic infrastructure remain barriers to routine implementation (Table 5) [155,156].

## 8. Clinical Translation and Stewardship

A mechanism-based stewardship approach is essential in the era of novel BL/BLI and siderophore β-lactams.

- It is important to match therapy to mechanism:
  ○ KPC (class A) producers typically respond well to CZA, M/V, or IMI/REL. However, KPC Ω-loop variants (e.g., D179Y/N) frequently emerge during CZA therapy, conferring resistance but restoring carbapenem susceptibility—a “seesaw effect.” Structural and in vitro data support reintroduction of meropenem or carbapenems guided by MIC changes [161].
  ○ MBL producers require ATM/AVI, uniquely stable to metallo-enzymes [26].
  ○ OXA-48-like producers often depend on good permeability; strains with porin loss may fail BL/BLI but remain cefiderocol-susceptible [162].
- When BL/BLI fails, the following should be considered:
  ○ Use M/V or IMI/REL if active in vitro; they may restore susceptibility in CZA-resistant isolates [30,163].
  ○ Cefiderocol is a key rescue agent, though high rates of heteroresistance demand vigilance.
  ○ For severe cases or documented heteroresistance, combination regimens, such as colistin or SUL-DUR with cefiderocol, are under evaluation [162].
- De-escalation opportunities:
  ○ In settings where KPC variants restore susceptibility, switching back to carbapenems when MICs return to susceptible levels may be safe and antibiotic-sparing.

### Combination Approaches in CRE/CRAB: Current Evidence and Stewardship Recommendations

Combination therapy is best justified when it addresses a specific mechanistic gap (e.g., MBL producers) or when clinicians face high-inoculum infection, limited source control, or suspected heteroresistance, rather than as routine empiric escalation [20]. In practice, combination regimens should be started for coverage of early uncertainty and then de-escalated once carbapenemase class and MIC stability are confirmed.

For MBL-producing CRE, the most supported β-lactam-based combination is CZA+ATM, leveraging aztreonam’s intrinsic stability to MBL hydrolysis while avibactam suppresses co-produced ESBL/AmpC/KPC activity. This approach is explicitly listed as a preferred option in the IDSA AMR Guidance (alongside cefiderocol monotherapy) for NDM/other MBL-producing Enterobacterales infections [20]. Clinical experience remains largely observational, but retrospective clinical series in VIM-producing infections report feasible use with clinical response signals, supporting its role as a practical, mechanism-driven option when dedicated agents are unavailable [163]. Stewardship recommendation: reserve CZA+ATM for confirmed or strongly suspected MBLs and reassess quickly as susceptibility and molecular results return, because unnecessary combination β-lactam exposure increases toxicity risk and selection pressure.

For CRAB, the pathogen-specific ATTACK phase 3 trial demonstrated the non-inferiority of SUL/DUR (with background imipenem–cilastatin) compared with colistin-based therapy and improved tolerability, supporting a sulbactam-centered backbone when available [39,164]. In contrast, randomized trials of polymyxin–carbapenem combinations failed to show outcome benefit over colistin alone, cautioning against routine combination use without a clear mechanistic rationale [165,166].

Where new agents are used, stewardship should favor optimized dosing and mechanism-aware selection over automatic combination. For example, FEP/TAN has phase 3 evidence in cUTI (CERTAIN-1) supporting clinical efficacy, but its MBL coverage is variant-dependent, and resistance concerns (e.g., NDM-9) justify continued surveillance rather than assuming universal MBL activity [167].

Practical stewardship recommendations across CRE/CRAB therefore include the following:

- start combination therapy only when it closes a mechanistic gap (MBL coverage; suspected heteroresistance near breakpoints),
- repeat AST and mechanism assignment when MICs are close to decision thresholds,
- de-escalate to the narrowest effective regimen once molecular/phenotypic concordance is established and source control is achieved.

## 9. Future Directions

Multidrug resistance, particularly carbapenem resistance, is increasingly driven by cumulative antibiotic exposure, invasive devices, and healthcare transmission, with CRE and CRAB now major contributors to therapeutic failure and excess mortality [168,169,170,171]. Emerging evidence further implicates the gut microbiome and device-associated infections as reservoirs and amplifiers of resistance determinants, highlighting that carbapenem resistance reflects a system-level failure extending beyond individual pathogens [172,173,174,175]. These dynamics have intensified carbapenem resistance in the post–COVID-19 era, as pandemic-related antibiotic overuse and healthcare strain accelerated the emergence and spread of resistant organisms [176,177,178,179].

The fight against carbapenem resistance is at a turning point, with several novel agents and diagnostic innovations emerging that may help to fill longstanding therapeutic gaps. Among the most promising is zosurabalpin (RG-6006), a first-in-class antibiotic targeting the lipopolysaccharide transporter of *A. baumannii*. Following encouraging preclinical and early clinical results, Roche has launched a Phase 3 trial specifically for CRAB infections, marking the first time in decades that an antibiotic with a truly novel mechanism of action is advancing toward regulatory approval [180]. Mechanistic studies confirm that zosurabalpin blocks the LptB_2_FGC complex, halting lipopolysaccharide transport and thereby crippling outer-membrane integrity in CRAB, with strong in vitro potency and efficacy in murine models [181,182].

Beyond zosurabalpin, the therapeutic pipeline is diversifying. A recent review catalogued more than a dozen antibiotics in active clinical development, alongside experimental non-traditional agents such as monoclonal antibodies (TRL-1068, CMTX-101), engineered peptides, microbiome modulators, and phage-based therapies [183]. These efforts reflect a recognition that multifaceted approaches will be required to address pathogens such as CRAB and MBL-producing Enterobacterales, which remain among the most therapeutically challenging. Additional compounds including cefepime–zidebactam, OMN6, and BV-100 are advancing through Phase II and III evaluation, with promising early activity against multidrug-resistant isolates [184].

Progress in treatment depends on equal advances in diagnostics. Same-day lateral flow immunoassays such as NG-TEST CARBA 5 have demonstrated excellent performance for detecting the most common carbapenemases in Enterobacterales, with one multicenter study reporting 100% positive agreement [185]. Yet performance varies across pathogens: assays optimized for Enterobacterales may underperform in *P. aeruginosa* or *Acinetobacter*. Comparative evaluations confirm that while the mCIM and its EDTA variant (eCIM) retain high sensitivity for KPC detection, sensitivity drops in some VIM-positive *Pseudomonas* strains, necessitating confirmatory orthogonal testing [186]. Likewise, Carba NP can miss unusual or low-level carbapenemase producers, leading to the importance of pairing phenotypic assays with molecular confirmation [187].

To further refine detection, surveillance strategies must incorporate molecular signatures such as carbapenemase gene amplification and porin L3 modifications, which have been linked to therapeutic failure and the spread of high-risk clones. Integrating these features into genomic epidemiology pipelines would improve the accuracy of resistance prediction and allow earlier recognition of deceptive susceptibility profiles [188].

At the therapeutic level, inhibitor development is another frontier. While agents such as avibactam and vaborbactam have improved outcomes against serine β-lactamases, they offer little coverage for OXA-48-like and MBL enzymes, which are increasingly prevalent in Enterobacterales and *Acinetobacter*. Dual-inhibitor concepts, combining boronate-based scaffolds with thiol- or zinc-binding pharmacophores, are being explored as a means to achieve broader coverage across enzyme classes [183]. Finally, there is a pressing need to standardize phenotypic proxies that correlate with mechanisms of resistance. The inoculum effect, where MICs shift dramatically with bacterial load, has been observed in isolates carrying amplified *bla*_KPC_ or *bla*_OXA_ genes. While long regarded as a laboratory artifact, emerging data suggest that this effect may predict treatment failure in high-burden infections [186]. Establishing reproducible assays to capture such phenomena, and linking them to genomic markers, would strengthen both clinical decision-making and the consistency of surveillance data across centers.

## 10. Conclusions

Carbapenem resistance has evolved from a primarily enzymatic phenomenon into a multilayered adaptive system encompassing β-lactamase diversification, gene amplification, plasmid dynamics, permeability remodeling, and efflux regulation. These mechanisms interact dynamically under antibiotic pressure, generating heteroresistance, inoculum effects, and fluctuating susceptibility patterns that challenge traditional diagnostic and therapeutic paradigms.

While the antimicrobial pipeline shows renewed momentum, significant gaps remain, particularly for MBL-producing Enterobacterales and globally disseminated CRAB. Diagnostic advances now permit rapid enzyme identification and increasingly sophisticated genomic interpretation, yet implementation disparities and access limitations threaten to widen global inequities in care.

Sustainable progress will require the integration of mechanism-aware diagnostics, quantitative genomic surveillance, optimized stewardship strategies, and equitable access to emerging therapies. Novel agents such as zosurabalpin and next-generation β-lactamase inhibitors offer cautious optimism, but their impact will depend on deployment within coordinated diagnostic-guided frameworks rather than empirical escalation.

Containing carbapenem resistance will therefore demand not only scientific innovation but also systemic reform, linking microbiology, clinical decision-making, public health surveillance, and global access initiatives. Only through such integrated, mechanism-informed strategies can the escalating threat of carbapenem-resistant pathogens be effectively mitigated.

## Figures and Tables

**Table 1:** Distribution, Evolution, and Clinical Impact of Major Carbapenemase Classes Across Gram-Negative Pathogens.

Pathogen	Predominant β-Lactamase Class	Key Variants/Evolution Since 2020	Prevalence in Patient Isolates (%)	Clinical Impact (%)	Key References
*Klebsiella pneumoniae* (CRKP)	Class A–KPC	>190 *bla*_KPC_ subtypes; Ω-loop D179Y/N mutations driving CZA resistance	41.2% bloodstream carbapenem resistance in WHO SE Asia; >25–40% in high-burden regions	30–50% 30-day mortality in CRKP BSI; 13.2% attributable mortality vs. susceptible strains (EURECA)	[4,61,62]
	Class D–OXA-48-like	Rising global prevalence; modest MIC elevation complicates detection	US prevalence increased from 0.3% (2019) to 8.2% (2021)	Associated with delayed detection and therapeutic escalation	[60]
*Acinetobacter baumannii* (CRAB)	Class D–OXA-23-like (dominant)	IC2 (ST2) ≈ 43.7% global clonal dominance	54.3% carbapenem resistance in bloodstream isolates globally	35–60% mortality in CRAB bacteraemia and ICU cohorts	[4,7,10]
	Class B–NDM (co-producers)	Plasmid-mediated spread; co-occurrence with OXA-23	Increasing reports of dual carbapenemase producers	Increased therapeutic failure risk	
*Pseudomonas aeruginosa* (CRPA)	Class B–VIM/IMP	VIM-92 on Tn1403 megaplasmid; environmental reservoirs (VIM-71)	Regional carbapenem resistance frequently 15–30% in invasive isolates	20–40% mortality in CRPA bacteraemia/VAP	[4,5]
	Class A–GES variants	Emerging carbapenem-hydrolyzing GES variants	Lower prevalence but under-detected	Often missed by routine panels	[4,61]
*Escherichia coli* (CRE subset)	Class B–NDM (NDM-1 to NDM-24)	M154L improves Zn binding; IncX3 plasmid spread of NDM-5	Annual resistance increase 12.5% (2018–2023, selected regions)	Increasing MBL-associated treatment failure	

**Table 2:** Practical laboratory algorithm for resistance mechanism inference when WGS is not available.

Step	Action	Interpretive Outcome	Key References
1. Initial screen	Test ertapenem MIC (most sensitive carbapenem)	Elevated ertapenem first suggests carbapenemase or porin deficiency	[100]
2. Confirmatory carbapenem	Add meropenem or imipenem MIC	Pattern helps stratify mechanism: ertapenem ≫ meropenem = permeability; uniform elevation = carbapenemase	[103]
3. Enzyme activity tests	Perform mCIM/eCIM, Carba NP, CIM variants, or inhibitor-based disks	Positive = assign to class A (KPC), B (MBL), D (OXA-48-like)	[101]
4. Molecular confirmation	Use multiplex PCR panels if available	Detects carbapenemase genes (KPC, NDM, OXA-48, etc.)	[90]
5. Discordant cases	If inoculum effect or heteroresistance present but enzyme tests negative	Consider porin loss (OmpK36) + ESBL/AmpC, or gene amplification	[96]
6. Clinical implication	Repeat AST/genomics during therapy; tailor therapy	Preserves mechanism-level resolution, guides empiric BL/BLI, cefiderocol, or alternative therapy	[101]

**Table 3:** Mechanisms Associated with Reduced Susceptibility to ATM/AVI.

Mechanism Category	Specific Alterations	Species Reported	Functional Effect	Key References
PBP3 structural modification	YRIK/YRIN 4-aa insertions	*E. coli*, *Enterobacter* spp.	Reduced aztreonam binding	[114]
Plasmid-mediated AmpC	CMY-42, CMY-141, CMY-145, CMY-146	*E. coli*	Increased β-lactam hydrolysis; elevated ATM/AVI MICs	[113]
AmpC overexpression/amplification	Increased plasmid copy number	*E. coli*, *Enterobacterales*	Enhanced resistance via higher enzyme dosage	[113]
ESBL + porin alteration	CTX-M + Omp defects	*Enterobacterales*	Reduced permeability + enzymatic hydrolysis	[29,115]
Multimechanistic convergence	PBP3 + β-lactamase + permeability	*E. cloacae*, *K. pneumoniae*	Synergistic resistance phenotype	[116]

**Table 4:** Mechanisms of resistance emerging under new carbapenem-sparing therapies.

Agent	Importance	Mechanisms Under Selection	Clinical Implications	Key References
Ceftazidime–avibactam (CZA)	First widely adopted KPC inhibitor; major impact on CRE treatment	KPC Ω-loop mutations (e.g., D179 variants, KPC-190, KPC-228); co-occurring OmpK36 loop 3 insertions/truncations; additional roles for efflux and alternative β-lactamases	Resistance can arise during therapy; some variants (Ω-loop mutants) paradoxically restore carbapenem susceptibility; repeat AST/genomic testing during treatment is recommended	[106,120,128]
Meropenem–vaborbactam (M/V) & Imipenem–relebactam (IMI/REL)	Designed to restore carbapenem activity against KPC	Porin disruption (OmpK35/36); increased *bla*_KPC_ copy number; less frequently KPC active-site changes	Cross-resistance between M/V and IMI/REL is common; failures typically linked to porin + gene dosage effects; testing for porin functionality and copy number is valuable for guiding salvage	[107,108,109,110]
Aztreonam–avibactam (ATM/AVI)	First BL/BLI with stable activity against MBL-producing CRE; FDA approval 2025 (Emblaveo)	PBP3 insertions (YRIK, YRIN motifs); hyperproduction of plasmid AmpC (e.g., CMY-42, CMY-145, CMY-146); ESBL overexpression; porin alterations; *bla*_NDM_ amplification	Resistance can emerge even before widespread rollout; close monitoring needed in MBL-endemic regions; CMY-positive *E. coli* particularly problematic	[115,116]
Cefiderocol	Siderophore cephalosporin with activity against CRE, CRPA, and CRAB; carbapenem-sparing	Mutations in siderophore receptors (*cirA*, *tonB*, *piuA*); β-lactamase context (NDM, PER, AmpC, KPC variants); efflux changes (e.g., *baeS*, *czcS*); PBP alterations (*mrcB*)	Multifactorial resistance, often emerging under therapy; heteroresistance reported in *A. baumannii* and *P. aeruginosa*; combination therapy may mitigate failures	[44,118,119,120,121]
Cefepime–taniborbactam (FEP/TAN)	Broad spectrum activity against Ambler A, C, D and many B carbapenemases; late-phase clinical trials	Reduced susceptibility with NDM variants (NDM-9, NDM-30, VIM-83); porin loss with ESBL/AmpC production; possible efflux contributions	Potent in vitro, but NDM evolution poses early warning; surveillance required prior to and after rollout	[123,124,125]
Sulbactam–durlobactam (SUL-DUR)	First FDA-approved (2023) agent specifically for CRAB; validated in ATTACK phase 3 trial	Overexpression of OXA carbapenemases (via ISAba1, esp. OXA-23/24/40); PBP3 (ftsI) mutations; efflux pump upregulation (AdeABC, AdeIJK); MBL production (resistance determinant)	Resistance already observed post-approval; risk of on-therapy selection; combination regimens (e.g., SUL-DUR + cefiderocol) under study	[129]

**Table 5:** Mechanism-oriented diagnostic workflow for Enterobacterales, *P. aeruginosa*, and *A. baumannii*.

Pathogen	Step 1: Rapid Screen	Step 2: Class Assignment/Confirmation	Step 3: Mechanism Refinement (When Results Are Discordant or Therapy Decisions Hinge on Details)	Notes/Pitfalls
Enterobacterales	mCIM on ertapenem/meropenem screen-positives or LFIA (e.g., NG-Test CARBA 5) for same-day rule-in.	eCIM to flag MBL; inhibitor discs (boronic acid for KPC/AmpC; EDTA/DPA for MBL) if mCIM weak/indeterminate; targeted PCR panel if available [144]	If enzyme tests negative but ertapenem ≫ meropenem MIC pattern: triage to porin loss + ESBL/AmpC. Consider porin immunoblot/PCR and AmpC/ESBL testing. If phenotype unstable or heteroresistant, quantify *bla* copy number (qPCR/ddPCR) or infer from WGS read-depth [157].	Carba NP may miss low-level OXA-48-like; pair with LFIA/PCR. MBL/KPC co-producers need eCIM + targeted molecular to avoid misclassification [140].
*Pseudomonas aeruginosa*	mCIM (lab-validated) or LFIA (colony) [100]	eCIM for MBL; inhibitor discs (EDTA); consider rapid flow-cytometry assays where available to differentiate KPC/MBL/OXA-48-like quickly [158]	If enzyme negative but carbapenem MICs high: evaluate OprD loss and efflux (screen via phenylalanine-arginine β-naphthylamide in research settings; regulator mutations require sequencing). For CZA/M/V/IMI-REL decisions, assess PER, VIM/NDM, and porin status [159].	Some Enterobacterales-optimized kits underperform in *P. aeruginosa*; confirm with orthogonal tests [100]
*Acinetobacter baumannii*	Species-tailored rapid assays from colonies or positive blood cultures to detect NDM/OXA carbapenemases [149].	If rapid test negative but carbapenem MICs high: perform targeted PCR for *bla***_OXA-23/24/58/NDM_**_;_ consider Carba NP only with caution (weak OXA hydrolysis) [139].	If enzyme present yet MICs unexpectedly high/variable: check for PBP3 (ftsI) changes, outer-membrane remodeling/efflux; for epidemiology and mechanism triage, use WGS [160].	Rapid Enterobacterales LFIAs may not cover Acinetobacter targets; use Acinetobacter-specific tests. Low-level OXA expression can evade colorimetric assays [139].

## Data Availability

No new data were created or analyzed in this study. Data sharing is not applicable to this article.
